# Supplementary material for: Quantitative Label-Free Single-Cell Proteomics on the Orbitrap Astral MS
Source: Mol Cell Proteomics. 2025 May 5;24(6):100982. doi: 10.1016/j.mcpro.2025.100982 (PMC12209951; doi:10.1016/j.mcpro.2025.100982)
Supplement: Supplemental data [file mmc1.docx]

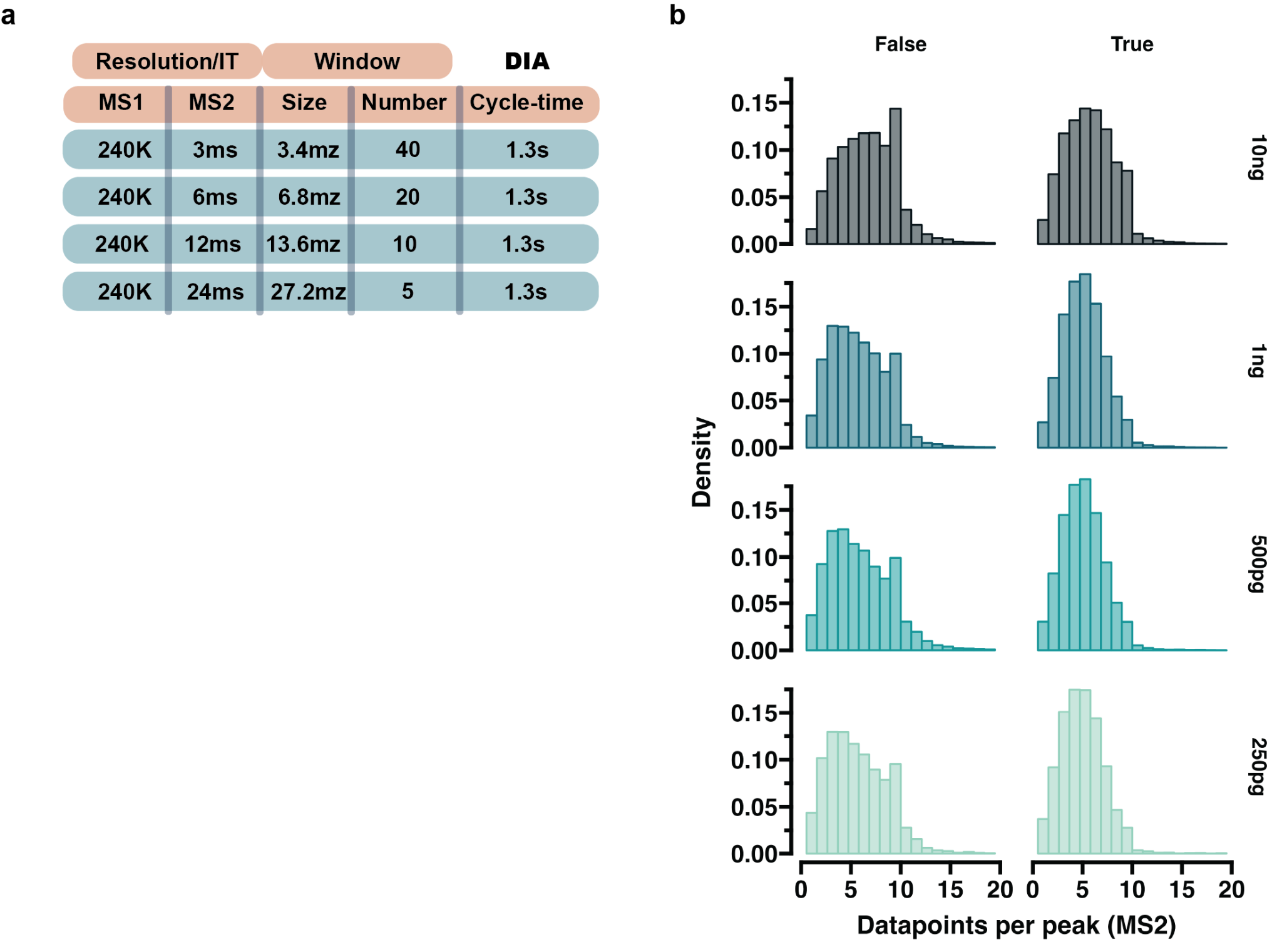


**Supplemental Figure 1. Scan cycle time for the different data acquisition method with and without FAIMS (Related to Figure 2). a)** Table outlining the injection times and isolation window sizes. MS1 was kept constant 100ms injection time. **b)** Histograms showing the data points per peak on MS2 level with the method from the table. Replicate 2 is specifically shown.
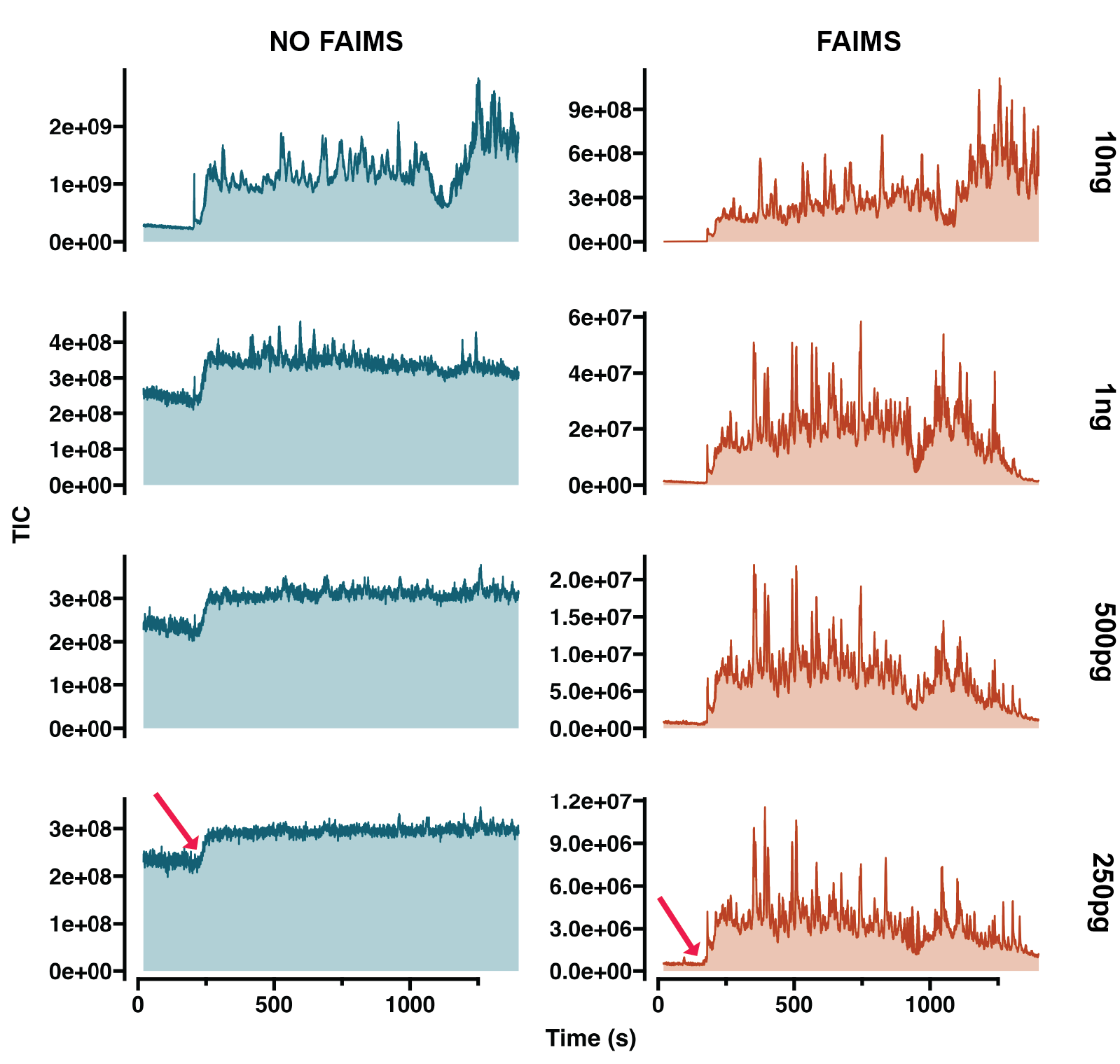


**Supplemental Figure 2. Chromatograms of total ion current (TIC) with and without FAIMS. a)** Chromatograms without FAIMS **b)** Chromatograms with FAIMS. Y-axis notes the TIC and X-axis time in seconds. The end part is truncated for easier visualization. Red arrows note the sample breakthrough point.


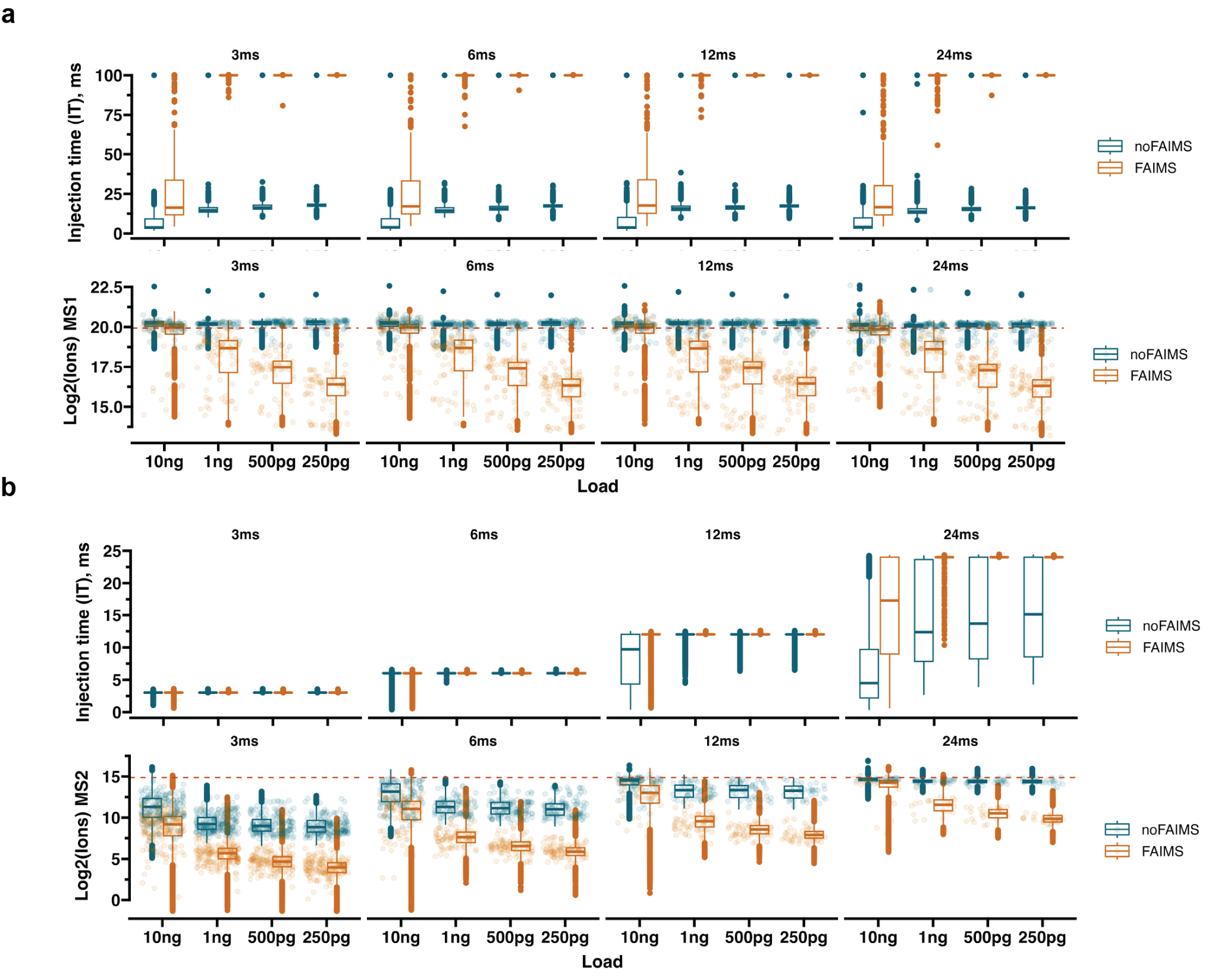


**Supplemental Figure 3. Injection time and ion count analysis with and without FAIMS. I**njection time and log2 transformed Ion count boxplots of MS1 level (Orbitrap) **a)**  and MS2 (ASTRAL) **b).** Colors note whether FAIMS was used. X-axis notes the input amount. Downsampled data points are added on top of the box to better represent the distribution.


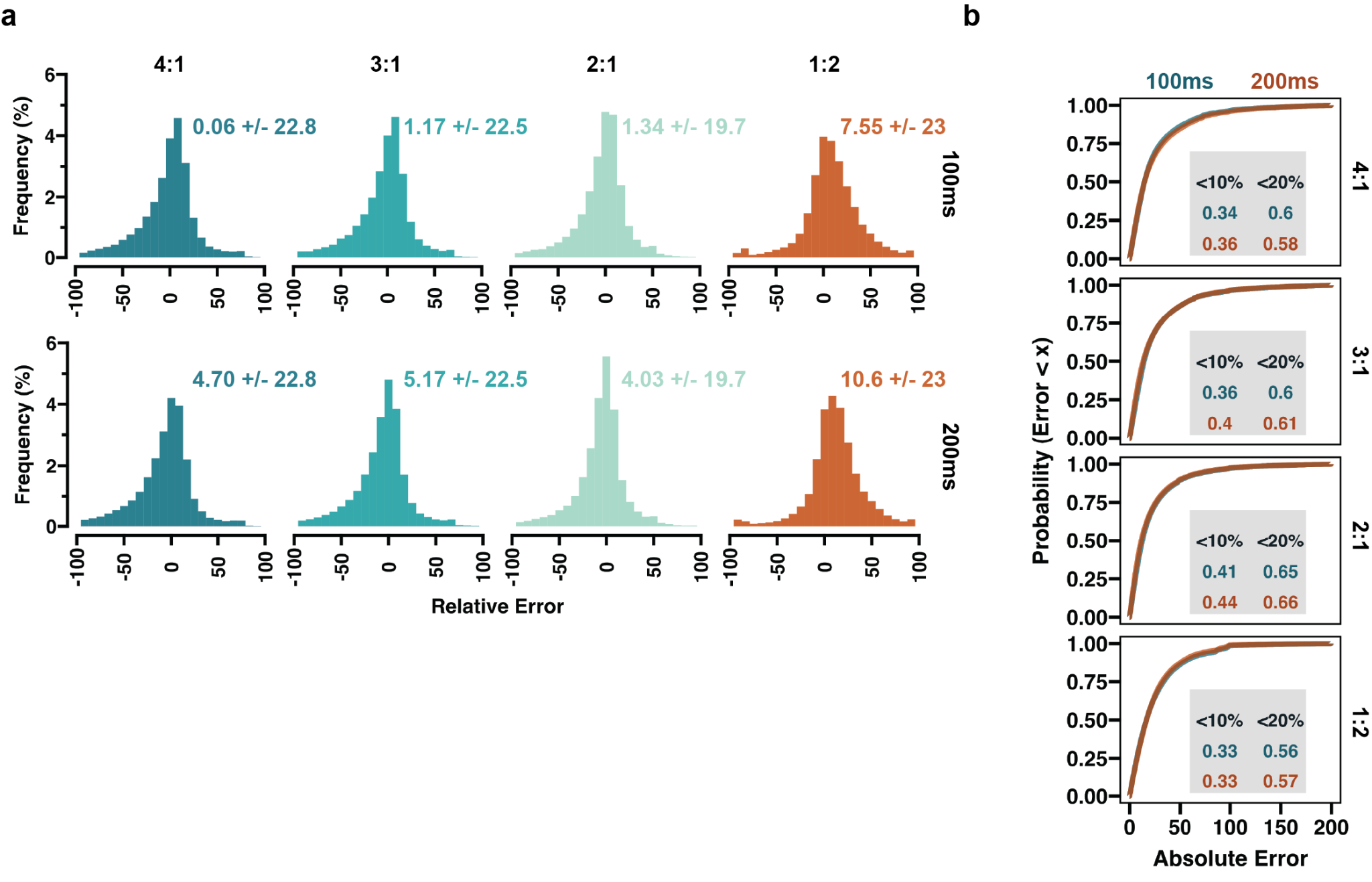


**Supplemental Figure 4. Relative error distribution on MS1 level with 100 and 200 ms injection time.** Hela peptide dilution ranging from 120pg to 1000pg was analyzed, 250pg was set as a reference and all the ratios are noted relative to this amount. 120pg-250pg is rounded to 1:2 for simplicity. **a)** Histogram of the relative error for all the tested ratios. Number notes the median error values and the median absolute deviation is given as the uncertainty on the number. **b)** Cumulative Distribution Function plots of the absolute error distribution for the different ratios. Color depicts the used injection time on MS1 level and the number represents the fraction of peptides below the noted error threshold.

**
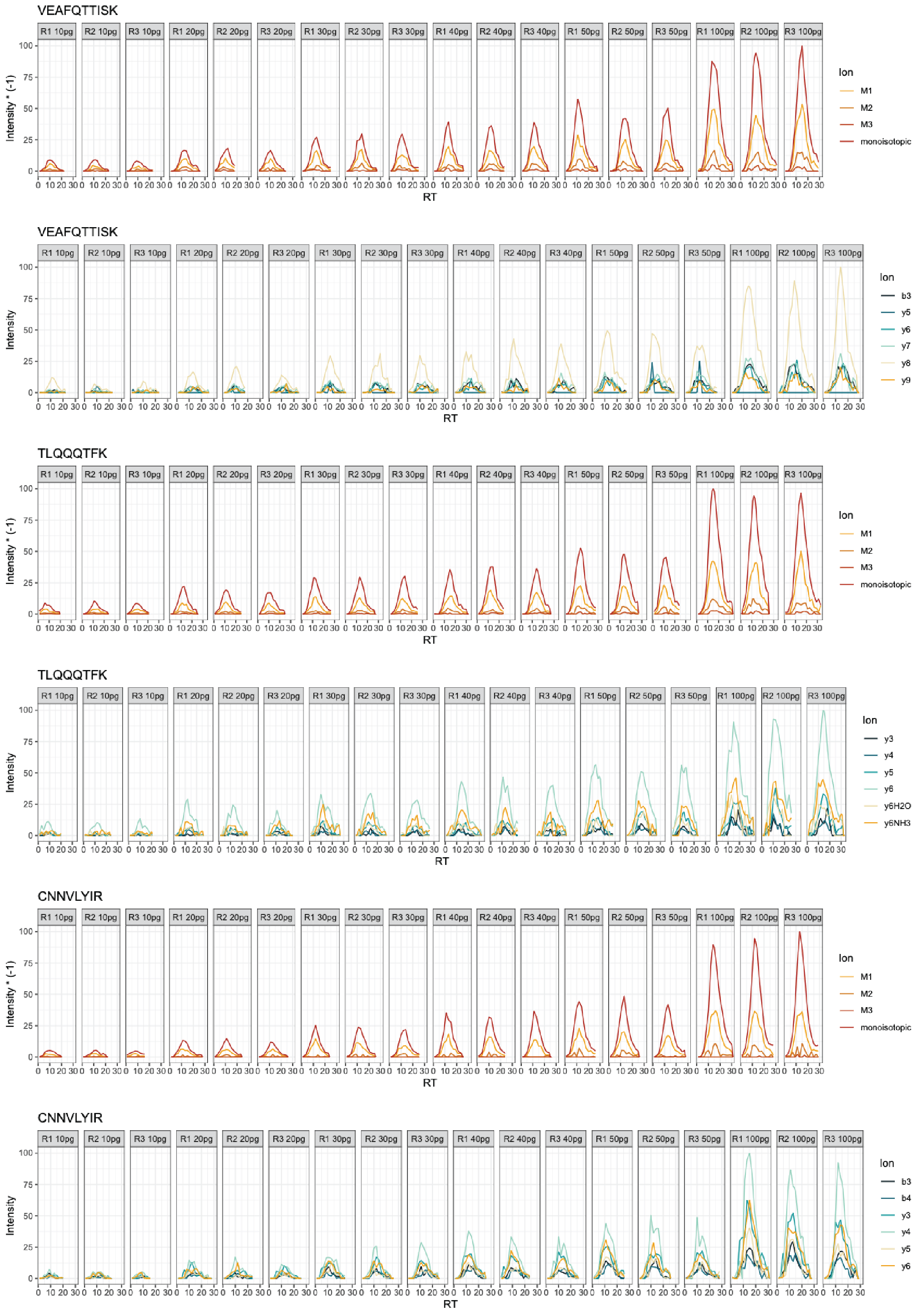

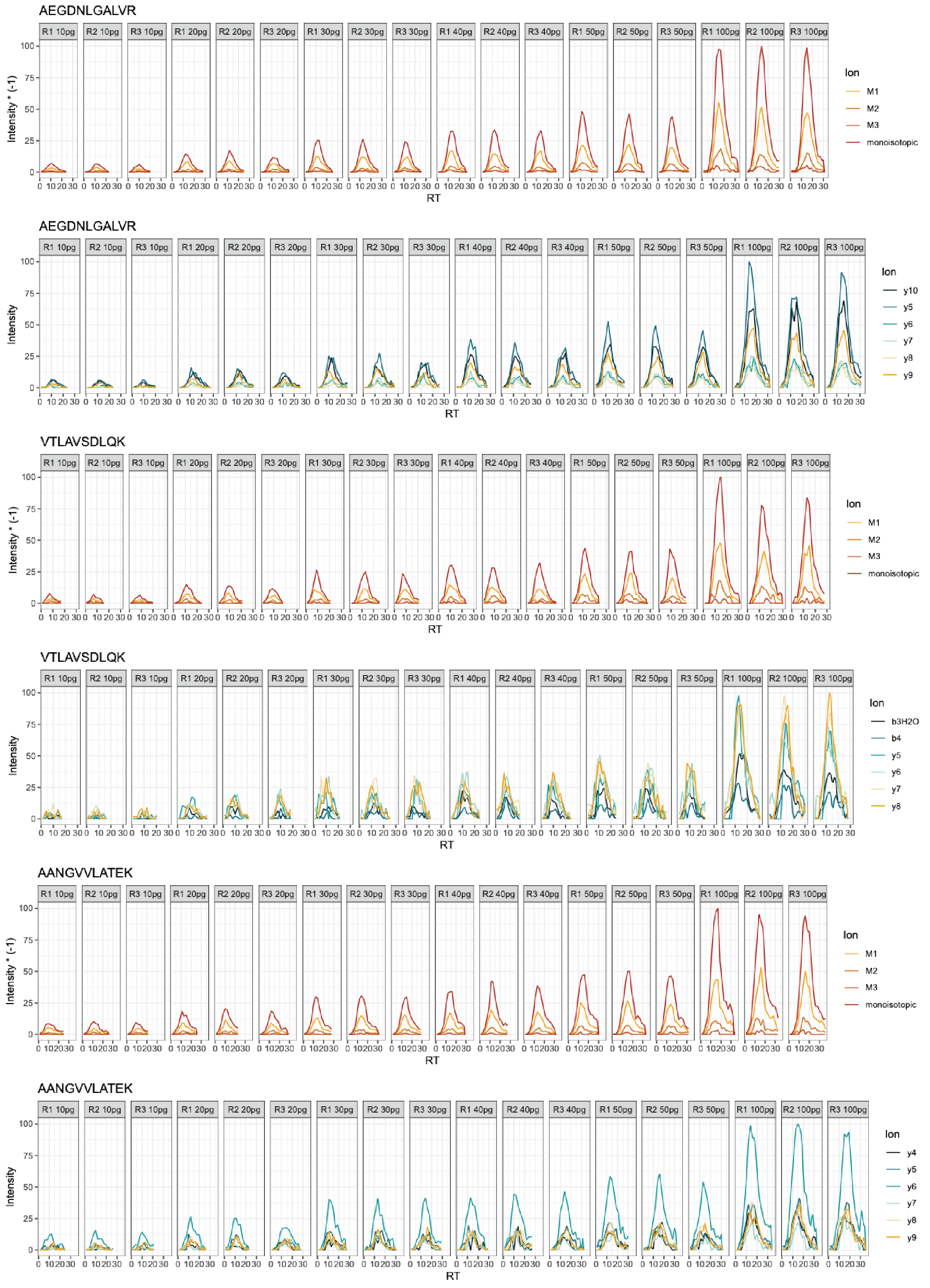
**

**Supplemental Figure 5. Extracted ion chromatograms of randomly selected peptides on precursors (MS1) and fragment (MS2) level.** The peptide amino acid sequence is indicated above each plot. Precursors isotope ions and fragment ions are indicated by different colors. The respective input is indicated in the grey box, y-axis notes the intensity and x arbitrary retention time.


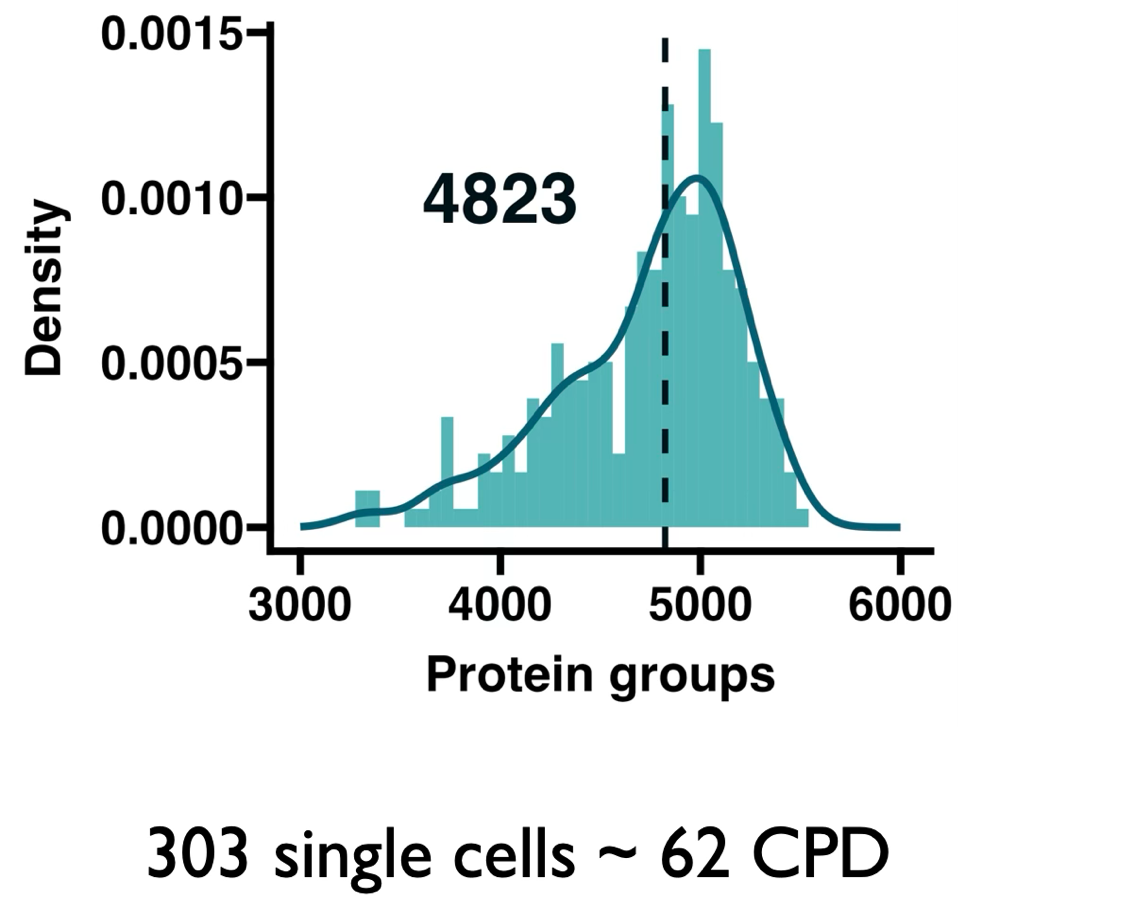


**Supplemental Figure 6. Per cell protein identification histogram of suspension HEK293 cells.** X-axis notes the protein groups, y the density of the distribution. Dashed line indicates the median identified protein number. Were we analyzed with a slightly different approach (See Methods and Experimental Design). A run 21 minute run to run method (-68SPD) was used and a lower injection time on MS2 level of 40ms.
